# Associations Among Sleep Duration, Physical Activity, and Nutrient Intake in Korean Adults

**DOI:** 10.3390/nu17142324

**Published:** 2025-07-15

**Authors:** Eunjae Lee, Seung-Taek Lim

**Affiliations:** 1 Institute of Sports & Arts Convergence (ISAC), Inha University, Incheon 22212, Republic of Korea; eunjaesports@gmail.com; 2 Waseda Institute for Sport Sciences, Waseda University, Saitama 341-0018, Japan; 3 College of General Education, Kookmin University, Seoul 02707, Republic of Korea

**Keywords:** sleep duration, physical activity, protein, vitamin D

## Abstract

Background/Objectives: This study aims to investigate the relationship between sleep duration, physical activity, and nutritional intake of calories and vitamins to determine the interconnections among sleep, physical activity, and dietary habits. Methods: Overall, 5491 participants (male = 2347, female = 3144) aged ≥ 18 years were recruited from the first survey of the 2023 9th Korea National Health and Nutrition Examination Survey (KNHANES). All participants were assessed for sleep duration, physical activity levels, and intake of vitamin D, carbohydrates, protein, and fat. Results: In both sexes, participants with ≥8 h of sleep per day had significantly higher levels of moderate-intensity physical activity (male: *p* = 0.026, female: *p* = 0.011), moderate-to-vigorous-intensity physical activity (male: *p* = 0.003, female: *p* = 0.004), vitamin D (male: *p* = 0.029, female: *p* = 0.008), protein (male: *p* < 0.001, female: *p* = 0.011), and fat (male: *p* = 0.007, female: *p* = 0.015) than those with < 8 h of sleep. In the unadjusted model, participants who did not meet the recommended protein intake were less likely to achieve sufficient levels of physical activity (OR = 1.59, 95% CI: 1.39–1.82) and adequate sleep duration (OR = 1.12, 95% CI: 1.10–1.16). Conclusions: Sleep duration, physical activity, and nutrient intake (particularly vitamin D and protein) appear to be interrelated. Therefore, increasing daily physical activity and ensuring adequate intake of protein and vitamin D is recommended to maintain healthy sleep duration.

## 1. Introduction

Sleep is a multidimensional construct encompassing various parameters that assess its quantity and quality [1]. Among these, sleep duration is closely linked to health outcomes. Distinguishing between sleep disruption and duration is crucial, as they represent distinct aspects of sleep. For instance, insomnia does not necessarily imply short sleep duration, although reduced sleep duration may indicate a more severe subtype of insomnia [2]. According to the National Sleep Foundation, an adequate sleep duration is 7–9 h for young and middle-aged adults (18–64 years) and 7–8 h for older adults (over 65 years) [3].

Studies show that habitual short sleep duration is linked to an increased risk of developing type 2 diabetes, even among individuals adhering to a healthy diet, as reported in a UK Biobank cohort study of adults aged 38–71 years [4]. A recent study reports that a combination of sufficient sleep, regular physical activity, and good nutrition—high-quality proteins and vitamins—are linked to reduced mortality [5]. Additionally, high consumption of processed foods, saturated fats, and added sugars correlates with poorer sleep quality and shorter sleep duration [6]. Poor sleep quality is associated with higher intake of added sugars and unsaturated fats, suggesting that poor sleep quality may contribute to increased food intake, reduced dietary quality, and elevated cardiovascular disease risk [7].

One study reports reduced physical activity in both adult males and females with insomnia and short sleep duration, with increases in weight, body mass index, and waist circumference, particularly among females [8]. Individuals with insufficient sleep also spent more time engaged in sedentary behaviors compared to those with adequate sleep [9]. In a previous study of 820 participants, leisure walkers were 34% more likely to report adequate sleep duration compared to non-leisure walkers, and those who met the recommendations for moderate or vigorous physical activity were more likely to have good sleep quality [10]. This relationship between sleep and physical activity has been linked to physical changes in young adults and older adults, as well as to adverse health outcomes. Physical activity, which is important for young adults and older adults, affects skeletal muscle, which is key to human metabolism [11]. Protein is an important factor in regulating muscle metabolism, and the interaction between post-workout metabolic processes and increased amino acid availability maximizes the stimulation of muscle protein synthesis [12]. These findings suggest that short sleep duration affects health and is also linked to physical activity and protein intake.

Sleep, physical activity, and nutrition are closely interrelated in a cyclical manner, but an understanding of the relationship between sleep duration, physical activity, and dietary intake remains lacking. A case–control study of sleep patterns in children with autism reveals mechanistic links between sleep architecture and nutrient metabolism that persist across the lifespan [13]. These studies should examine multiple behavioral goals rather than a single exposure. In addition, barely any studies have investigated the relationship between sleep duration, physical activity, and nutrient intake in the Korean population. The hypothesis of this study is that shorter sleep duration will lead to a decrease in physical activity and a decrease in the quality of nutrient intake. Therefore, this study aims to investigate how variations in sleep duration affect physical activity and nutritional intake of calories and vitamins, with the goal of elucidating the interrelationships among sleep, physical activity, and dietary patterns. Understanding how sleep deprivation affects physical activity and nutrient intake could provide a scientific basis for healthy lifestyle behaviors.

## 2. Methods

### 2.1. Participation

**Figure 1:**
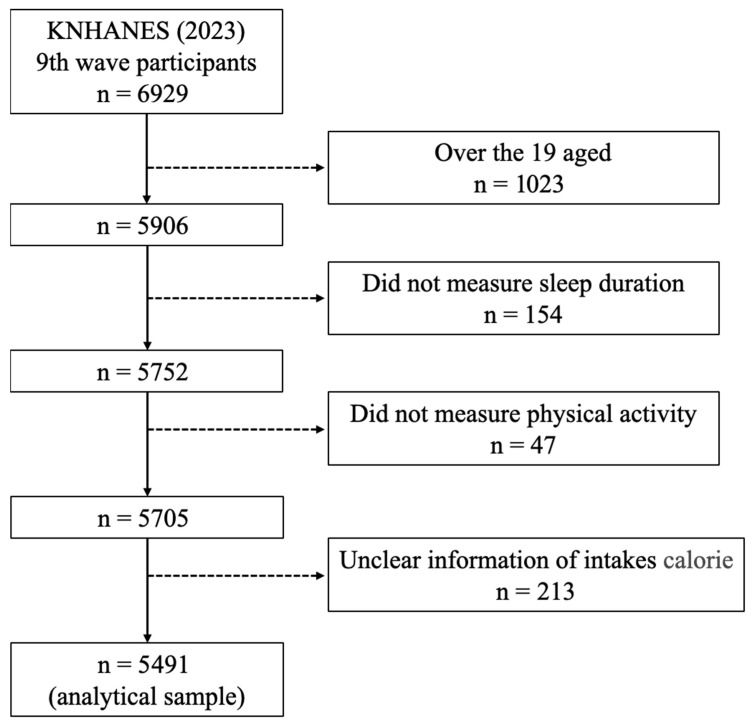
Flow chart of the study sample selection.

The data used for these analyses were obtained from the 2023 9th Korea National Health and Nutrition Examination Survey (KNHANES). Participants were selected randomly using a multistage, stratified probability sampling method for selecting household units based on geographic regions, encompassing both urban and rural areas. The present study included 5491 participants aged ≥ 18 years (male = 2347, female = 3144, age range 18~80 years), excluding those with missing data on sleep duration, physical activity, and calorie intake (Figure 1).

The 9th KNHANES was approved by the Institutional Review Board of the Korea Disease Control and Prevention Agency (KDCA) (2022-11-16-R-A) [14]. Written informed consent was obtained from all participants as part of the original survey. Raw data were sourced through official procedures from the KNHANES website under the KDCA, and all personal data were fully anonymized.

Table 1 presents the physical characteristics of participants.

### 2.2. Sleep Duration

Sleep duration was assessed using self-reported responses to the question, “How many hours do you sleep per day?” Participants reported their sleep duration separately for weekdays (or workdays) and weekends (or non-workdays). Average daily sleep duration was calculated by combining the two questions and dividing by two.

Responses were categorized into two groups (≥8 h per day and <8 h per day) based on sleep duration recommendations for older adults by the National Sleep Foundation and the Centers for Disease Control and Prevention [3,15].

### 2.3. Level of Physical Activity

This study applied the computerized Korean version of the International Physical Activity Questionnaire (IPAQ), specifically the long self-administered format, for a typical week, as outlined in the IPAQ operations manual. The 7-item IPAQ assessed total minutes of moderate-to-vigorous-intensity physical activity (MVPA) over the previous seven days. It collected detailed information on the duration (frequency and average length per session) of walking, moderate-intensity physical activity (MPA), vigorous-intensity physical activity (VPA), and sedentary behavior (weekday and weekend sitting time). To improve accuracy, the questionnaire included specific examples of moderate and vigorous physical activities. Responses were aggregated for each category (VPA, MPA, and MVPA) to calculate the total weekly duration of physical activity [16].

### 2.4. Assessment of Vitamin D, Carbohydrate, Protein, and Fat Intake

Nutrient intake, such as dietary carbohydrates, proteins, and fats, was assessed following the KNHANES protocol. Food intake over 24 h was estimated through individual dietary recall interviews conducted during household visits. The nutrient intake from food alone, excluding dietary supplements, was calculated. Nutrient intake was calculated as the sum of all food and nutrients consumed by one individual within 1 day [17].

Investigators manually recorded all food intake by participants the previous day on a standardized survey sheet. Tertiary food codenames were assigned to convert reported food into nutrient values. The analyses focused on vitamin D, carbohydrate, protein, and fat intake.

### 2.5. Statistical Analysis

Data are presented as mean ± standard deviation. Analyses were performed using SPSS version 29.0 (SPSS Inc., Chicago, IL, USA). Binary logistic regression analyses were conducted to examine the independent and combined relationship of protein intake with physical activity and sleep duration. Odds ratios (ORs) and 95% CIs were calculated. The reference group for the joint association analysis comprised individuals meeting the Dietary Reference Intakes for Koreans (KDRIs) protein guidelines (65 g/day for males and 55 g/day for females aged ≥ 18 years) [18]. The adjusted models controlled for covariates including aged (18–64 aged and ≥65 aged), sex (male, female), body mass index (<25, ≥25), waist circumference (male: <85 cm, ≥85; female: <90 cm, ≥90), smoking status (smoker, non-smoker), and alcohol consumption (drinker, non-drinker). Participants were stratified by sleep duration (≥8 h/day vs. <8 h/day), and group differences in physical activity and nutrient intake (vitamin D, carbohydrate, protein, and fat) were further analyzed using an independent *t*-test. Pearson’s correlation coefficients were calculated to assess relationships among sleep duration, protein intake, physical activity, and vitamin D. Statistical significance was set at *p* < 0.05.

## 3. Results

### 3.1. Physical Activity Levels

Table 2 presents physical activity levels by sex and sleep duration group.

Among males, those with ≥8 h of sleep per day reported significantly higher levels of MPA (*p* = 0.026), VPA (*p* = 0.010), and MVPA (*p* = 0.003) than those with <8 h of sleep per day. In females, MPA (*p* = 0.011) and MVPA (*p* = 0.004) were significantly higher in the ≥8 h sleep group. However, VPA did not differ significantly in female groups.

### 3.2. Vitamin D, Carbohydrate, Protein, and Fat Intake

Table 3 presents nutrient intake by sex and sleep duration group.

In the male group, the ≥8 h sleep group had significantly higher intakes of vitamin D (*p* = 0.029), protein (*p* < 0.001), and fat (*p* = 0.007) than the <8 h group. However, carbohydrates did not differ significantly. In the female group, intakes of vitamin D (*p* = 0.008), protein (*p* = 0.011), and fat (*p* = 0.015) were significantly higher in the ≥8 h sleep group. However, carbohydrates showed no significant difference.

### 3.3. Relationship Between Protein Intake, Physical Activity, and Sleep Duration

Table 4 presents the independent relationship between protein intake, physical activity, and sleep duration.

In the unadjusted model, participants who did not meet the recommended protein intake (OR = 1.59, 95% CI: 1.39–1.82) were more likely to engage in insufficient levels of physical activity. After adjusting for covariates, including sex, body mass index, waist circumference, smoking status, and alcohol consumption, the relationship remained significant but was slightly attenuated (OR = 1.35, 95% CI: 1.17–1.55). This suggests that adults with inadequate protein intake were 1.35 times more likely to experience lower levels of physical activity.

In the unadjusted model, participants who did not meet the recommended protein intake (OR = 1.12, 95% CI: 1.10–1.16) were less likely to achieve sufficient sleep duration. After adjusting for covariates, including sex, body mass index, waist circumference, smoking status, and alcohol consumption, the relationship was slightly attenuated but remained significant (OR = 1.06, 95% CI: 1.01–1.10). This indicates that adults with inadequate protein intake were 1.06 times more likely to experience reduced sleep duration.

### 3.4. Correlation Coefficients Between Sleep Duration and Physical Activity, Protein, and Vitamin D

**Figure 2:**
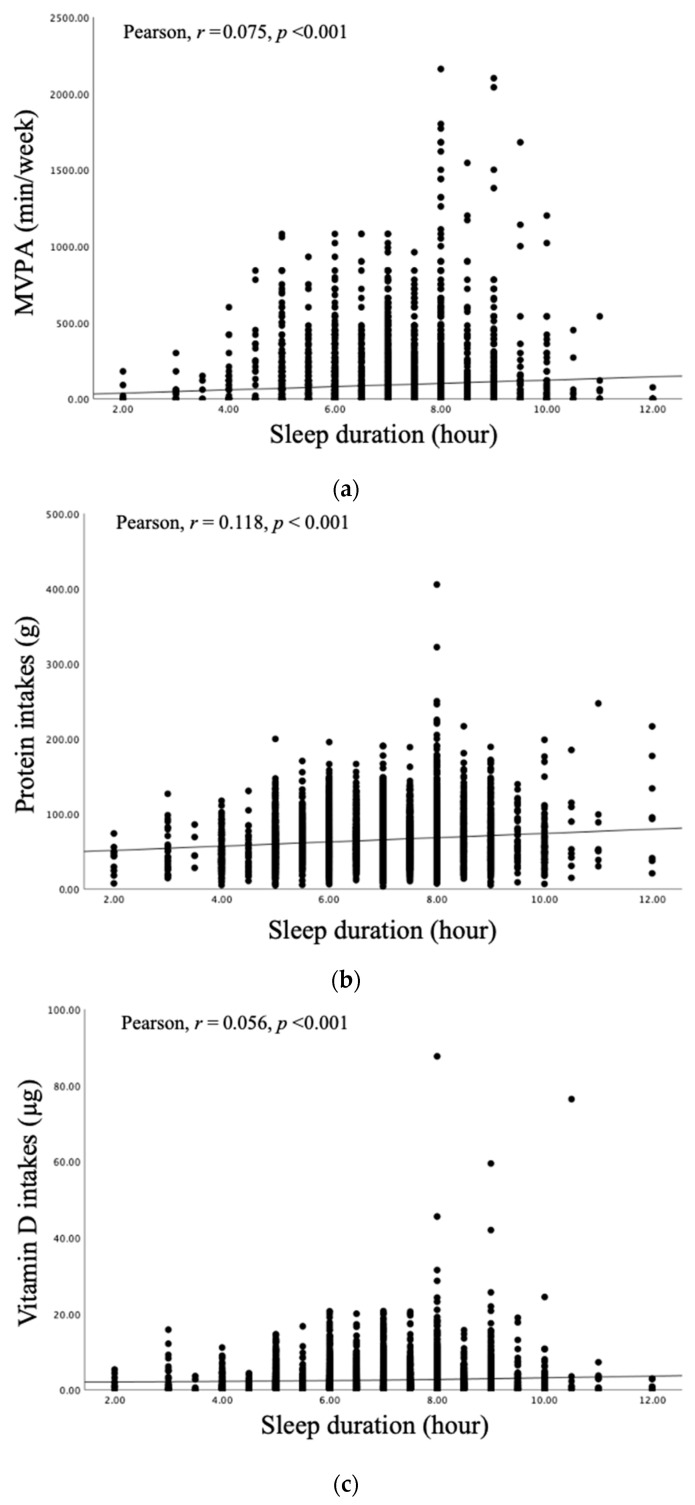
Pearson’s correlation coefficients between sleep duration and physical activity, protein, and vitamin D. (**a**) Sleep duration with MVPA; (**b**) Sleep duration with protein intake; (**c**) Sleep duration with vitamin D intake.

Figure 2 presents the correlation coefficients between sleep duration and physical activity, protein intake, and vitamin D intake. A positive correlation was observed between sleep duration and MVPA (*r* = 0.075, *p* < 0.001) (Figure 2a), protein intake (*r* = 0.118, *p* < 0.001) (Figure 2b), and vitamin D intake (*r* = 0.056, *p* < 0.001) (Figure 2c) across all participants.

## 4. Discussion

This study showed that Korean adults who did not meet the recommended protein intake exhibited approximately 1.59 times lower levels of physical activity and 1.12 times shorter sleep duration than those who met the recommended protein intake. In both sexes, individuals in the ≥8 h per day sleep duration group had significantly higher intakes of vitamin D, protein, and fat than those with <8 h of sleep. Similarly, levels of MPA and MVPA were significantly higher in the ≥8 h sleep group than in the <8 h sleep group. Positive correlations were observed, indicating that sleep duration increased as MVPA, protein intake, and vitamin D intake increased.

Short sleep duration, a key characteristic of sleep and certain psychiatric disorders, is a growing concern in modern society [19]. Short sleep duration is potentially linked to social factors, such as intense work tasks and unhealthy lifestyle behaviors [20]. A 10-year follow-up study of 8958 participants aged 50–95 years shows that low physical activity and suboptimal sleep independently contribute to cognitive decline. Short sleep is related to accelerated cognitive decline, while participants with high physical activity and optimal sleep demonstrate higher cognitive performance than participants in all other combinations of lower physical activity and sleep duration [21]. A 7-year follow-up study of average 62-year-old adults reports that shorter sleep duration (<6 h/day) is related to an increased risk of type 2 diabetes compared to that of normal sleep duration (HR = 1.21, 95% CI: 1.03–1.41). This increased risk appeared to be mitigated by physical activity [22]. Among college students, increasing physical activity and ensuring adequate sleep are reported as key health promotion strategies because of their beneficial effects on quality of life [23]. The present study reported that both males and females in the ≥8 h sleep group engaged in significantly higher MPA and MVPA than those in the <8 h group. For VPA, a significant difference was observed in males but not in females (Table 2). A positive correlation was also observed between sleep duration and MVPA (Figure 2a). The study shows that the combined effects of short sleep duration and moderate or low physical activity are greater than the effects of each considered independently, suggesting a synergistic effect. The estimated additive effect of short sleep duration and moderate or low physical activity exceeds that of high physical activity alone [24]. In females, resting energy expenditure is affected by hormonal changes before and after menopause, and the increase in the sympathetic nerve branch plays an important role [25]. Phytoestrogens, the female sex hormones, can have a positive effect on muscle mass due to their estrogenic properties [25]. Estrogenic α and β are expressed and localized within skeletal muscle tissue and tendons and ligaments, suggesting a direct effect of estrogen [26]. Furthermore, the absence of a significant difference in VPA among females in this study is a reflection of cultural norms in Korea, where males are expected to perform strenuous outdoor labor, while females are more often engaged in domestic tasks.

Sleep duration also influences nutrient imbalance. One study reports that individuals with short sleep duration (<5 h) consumed less protein, carbohydrates, sugars, fiber, and total fat than normal sleepers [27]. A study of elderly patients with obesity also reports a negative correlation between sleep duration and intakes of monounsaturated fatty acids, dietary cholesterol, and total protein [28]. Furthermore, individuals with <6 h sleep duration were twice as likely to have serum 25 (OH) D < 20 ng/mL compared to those who sleep 6 h or more, even after adjusting for confounding variables. Males were four times more likely to have 25 (OH) D < 20 ng/mL, while no difference was observed in females [29]. The present study showed that both male and female participants in the ≥8 h sleep duration group had significantly higher intakes of vitamin D, protein, and fat than those in the <8 h sleep group. However, carbohydrate intake did not differ significantly between groups. Protein intake is particularly related to advancing age. In a study of older adults, participants who met the recommended protein intake had significantly higher muscle mass (OR = 2.16) and strength (OR = 2.31) than those who did not [17]. Additionally, a study of 104 healthy adults aged 50–75 years reports a positive correlation between the tryptophan-to-large neutral amino acid ratio and sleep duration. Among middle-aged and older adults, sleep duration was also positively correlated with dietary protein intake [30]. Furthermore, individuals who did not meet the recommended protein intake were 1.59 times more likely to have reduced physical activity and 1.12 times more likely to have shorter sleep duration. These findings suggest that adequate protein intake will be effective in improving sleep duration and physical activity. A positive correlation was also observed between sleep duration and protein intake (Figure 2b). Most studies linking sleep and fat focus on obesity. This study showed that both male and female participants in the ≥8 h sleep group had significantly higher fat intake than those in the <8 h group. Although the obesity status of participants was not assessed, the observed differences may be related to the level of physical activity. Basal metabolic rate (5043 ± 548 kJ/day in women and 6213 ± 656 kJ/day in men), total daily energy expenditure measured by doubly labeled water (8372 ± 1324 kJ/day and 11,453 ± 1834 kJ/day), and physical activity level (1.66 ± 0.17 and 1.85 ± 0.30, respectively) were significantly higher in men [31]. This elevated energy expenditure suggests a potential need for nutritional supplementation. Carbohydrate intake appears to have a limited effect in Korea, where rice is the staple food. A recent study using the Pittsburgh Sleep Quality Index shows that vitamin D supplementation reduced sleep latency and increased sleep duration in middle-aged adults [32]. Furthermore, a meta-analysis reports that vitamin D deficiency is related to a higher risk of sleep disturbances, including poor sleep quality, short sleep duration, and daytime sleepiness [33]. Vitamin D may influence sleep regulation through its role in melatonin synthesis [34]. Vitamin D receptors have been identified in brain regions that directly or indirectly influence sleep in humans [35]. In this study, both males and females in the ≥8 h per day sleep group had significantly higher vitamin D intake than those in the <8 h per day group. A positive correlation was also observed between sleep duration and vitamin D intake (Figure 2c). These findings suggest that adequate protein and vitamin D intake may be associated with healthier sleep duration patterns, meriting further investigation through prospective or interventional studies.

The present study has some limitations. First, although shorter sleep duration is problematic, long sleep duration might be also causing health problems. This study categorized participants based on the recommended 8 h of sleep, but future studies should explore a broader range of sleep durations. Second, the nutrient analysis requires greater specificity. For instance, protein intake should be differentiated by source (animal vs. plant) [36], and carbohydrates should be classified by type (monosaccharides, disaccharides, and polysaccharides) [37]. Future studies should examine health status. For example, participants with obesity may have different nutritional requirements, and those with osteoporosis may exhibit altered vitamin D metabolism and needs. Additionally, comorbidities that may affect sleep duration include depression, chronic illnesses, and sleep disorders (obstructive sleep apnea). Furthermore, intake may vary depending on the financial ability to purchase protein foods. Therefore, future studies should consider the health condition and financial status of participants. Finally, survey measures of diet, sleep duration, and physical activity can lead to potential misclassification bias due to intra-individual variability and recall bias. Future research should analyze this using objective measurement tools.

## 5. Conclusions

This study provides robust evidence that Korean adults who did not meet the recommended protein intakes had approximately 1.59 times lower levels of physical activity and 1.12 times shorter sleep duration than those who met the recommendations. Furthermore, individuals with ≥8 h of sleep per day demonstrated significantly higher levels of MPA, MVPA, vitamin D, protein, and fat intake than those with <8 h sleep per day. These findings suggest a close interrelationship among sleep duration, physical activity, and nutrient intake (particularly vitamin D and protein). Therefore, these findings suggest that adequate protein and vitamin D intake may be associated with healthier sleep duration patterns, meriting further investigation through prospective or interventional studies.

## Figures and Tables

**Table 1:** The characteristics of the participants.

Variable	Male (*n* = 2347)		Female (*n* = 3144)	
	≥8 h (*n* = 776)	<8 h (*n* = 1571)	≥8 h (*n* = 927)	<8 h (*n* = 2217)
Age (years)	51.92 ± 18.18	54.73 ± 16.06	48.46 ± 17.16	56.04 ± 15.45
Height (cm)	170.89 ± 6.80	170.43 ± 6.75	157.39 ± 6.32	157.37 ± 6.33
Weight (kg)	71.70 ± 12.97	71.62 ± 12.27	58.83 ± 10.42	58.64 ± 9.76
BMI (kg/m^2^)	24.47 ± 3.62	24.58 ± 3.46	23.35 ± 3.91	23.67 ± 3.68
WC (cm)	88.00 ± 9.95	88.24 ± 9.60	79.53 ± 10.87	81.18 ± 10.15
SBP (mmHg)	122.07 ± 13.53	121.67 ± 14.83	114.77 ± 16.50	118.49 ± 16.36
DBP (mmHg)	75.99 ± 9.35	75.62 ± 9.50	71.70 ± 9.34	72.55 ± 9.00
Sleep duration (h)	8.47 ± 0.72	6.43 ± 0.90	8.78 ± 0.58	6.27 ± 1.03

BMI, body mass index; WC, waist circumference; SBP, systolic blood pressure; DBP, diastolic blood pressure.

**Table 2:** Physical activity levels of each group by sleep duration and sex.

Variable	Male (*n* = 2347)		*p*-Value	Female (*n* = 3144)		*p*-Value
	≥8 h (*n* = 776)	<8 h (*n* = 1571)		≥8 h (*n* = 927)	<8 h (*n* = 2217)	
MPA (min/week)	102.33 ± 220.3	82.17 ± 152.90	0.026	71.96 ± 186.67	57.53 ± 123.00	0.011
VPA (min/week)	29.89 ± 109.94	21.33 ± 74.33	0.010	14.06 ± 61.36	10.48 ± 43.86	0.066
MVPA (min/week)	132.22 ± 269.29	103.50 ± 184.97	0.003	86.02 ± 204.02	68.01 ± 135.86	0.004

MPA: moderate physical activity, VPA: vigorous physical activity, MVPA: moderate-to-vigorous physical activity.

**Table 3:** Nutrient intake by sleep duration and sex.

Variable	Male (*n* = 2347)		*p*-Value	Female (*n* = 3144)		*p*-Value
	≥8 h (*n* = 776)	<8 h (*n* = 1571)		≥8 h (*n* = 927)	<8 h (*n* = 2217)	
Vitamin D (µg)	3.13 ± 5.21	2.76 ± 3.17	0.029	2.63 ± 4.42	2.28 ± 2.80	0.008
Carbohydrates (g)	285.86 ± 113.28	285.53 ± 104.36	0.944	228.19 ± 96.37	227.29 ± 92.33	0.806
Protein (g)	79.13 ± 40.21	73.18 ± 28.54	<0.001	60.32 ± 29.74	57.52 ± 27.66	0.011
Fats (g)	55.20 ± 42.86	50.89 ± 32.29	0.007	43.76 ± 28.40	41.08 ± 28.13	0.015

**Table 4:** Independent relationships of objectively measured protein intake with physical activity and sleep duration.

	Unadjusted		Adjusted ^a^	
	OR (95% CI)	*p*-Value	OR (95% CI)	*p*-Value
Physical activity Protein intake below KDRIs Protein intake above the KDRIs	1.00 1.59 (1.388–1.823)	<0.001	1.00 1.35 (1.169–1.554)	<0.001
Sleep duration Protein intake below KDRIs Protein intake above the KDRIs	1.00 1.12 (1.070–1.162)	<0.001	1.00 1.06 (1.010–1.103)	<0.001

OR: odds ratio, CI: confidence interval. Total of 150 min or more of physical activity (*n* = 1170), less than 150 min of physical activity, (*n* = 4321). Recommended protein intake (*n* = 3243), not recommended protein intake (*n* = 2248). ^a^ Adjusted for age (18–64 aged and ≥65 aged), sex (male and female), body mass index (<25 and ≥25), waist circumference (male, <85 cm and ≥85; female, <90 cm and ≥90), smoking status (smoker and non-smoker), and alcohol consumption (drinkers and non-drinkers).

## Data Availability

Raw data were sourced through official procedures from the KNHANES website under the KDCA, and all personal data were fully anonymized.
